# Acute stroke in persons 85 years or older—clinical characteristics, impact of frailty, and predictors of outcome

**DOI:** 10.3389/fneur.2025.1689225

**Published:** 2025-11-04

**Authors:** Johan Sanner, Bente Thommessen, Mia von Euler, Jakob O. Ström, Brynjar Fure

**Affiliations:** ^1^Faculty of Medicine and Health, School of Medical Sciences, Örebro University, Örebro, Sweden; ^2^Department of Neurology and Rehabilitation, Central Hospital Karlstad, Karlstad, Sweden; ^3^Department of Neurology, Division of Medicine, Akershus University Hospital, Lorenskog, Norway; ^4^Sophiahemmet University, Stockholm, Sweden; ^5^Department of Neurology, Faculty of Medicine and Health, School of Medical Sciences, Örebro University, Örebro, Sweden; ^6^Department of Internal Medicine, Central Hospital, Karlstad, Sweden

**Keywords:** stroke, older, outcome, frailty, predictors

## Abstract

**Introduction:**

Stroke cases among older people will increase due to demographic changes, necessitating an optimization of care strategies for this population. We aimed to examine clinical characteristics, prognostic factors, and the impact of frailty in an older stroke cohort.

**Patients and methods:**

We consecutively included 120 patients aged ≥85 years with acute ischemic or hemorrhagic stroke at a regional hospital in Sweden. Baseline characteristics, etiological subclassification, National Institutes of Health Stroke Scale (NIHSS) on admission, pre- and post-stroke functional level and frailty, measured by the modified Rankin Scale (mRS), the Barthel ADL Index (BI), and the Clinical Frailty Scale (CFS), were recorded. Regression analyses were conducted to evaluate predictors of death or poor functional outcome, defined as an mRS of ≥4, at 3 months.

**Results:**

The mean age was 89.1 years and 57.5% were women. Ischemic strokes accounted for 90.8% and atrial fibrillation was diagnosed in 55.8%. Overall, 26.6% received reperfusion therapy. At 3 months, the mortality and poor functional outcomes were 35.8 and 54.2%, respectively. A multivariate regression analysis identified age ≥89 years, BI ≤70, CFS ≥ 7 prior to stroke, total anterior circulation infarction, NIHSS ≥15 on admission, and post-stroke dysphagia as independent predictors of death or poor outcome.

**Discussion and conclusion:**

We found a high prevalence of cardioembolic disease in older people with stroke, emphasizing the importance of diagnosing atrial fibrillation and optimizing anticoagulant treatment. In addition to well-known predictors, severe frailty prior to stroke and post-stroke dysphagia predicted poor outcome. In the growing older stroke population, assessment of frailty may be beneficial in decision-making regarding interventions and direction of care.

## 1 Introduction

Increased life expectancy is likely to triple the global population aged above 80 years between 2020 and 2050 (1). The risk of stroke increases dramatically with age, doubling with each decade beyond 55 years (2). Thus, even though the stroke incidence is decreasing among older persons in many Western countries, the number of stroke cases is expected to increase substantially due to the demographic shift (3).

Stroke remains a common cause of death and disability, and the outcome is highly age-dependent. In older people, mortality and the risk of disability are elevated due to pre-existing impairments, multiple comorbidities, and often more severe strokes (4–6). However, there is a wide variation in the health status and autonomy of older persons. While many older individuals with multiple diseases are dependent and require considerable care, others have a high quality of life and a healthy life expectancy of many years (7–9).

Etiology and vascular risk factors of stroke in older persons seem to differ from those in younger populations. In advanced age, the importance of hypertension, diabetes, smoking, and hyperlipidemia appears to be diminished, whereas atrial fibrillation (AF), leading to cardioembolic ischemic stroke, emerges as a major risk factor (10, 11). In hemorrhagic stroke, cerebral amyloid angiopathy and anticoagulation-related hemorrhage are common causes (12).

Given the diversity among older individuals, the risk of harmful side effects, drug–drug interactions, and overtreatment, it may be inappropriate to adhere strictly to standardized stroke guidelines, both for prevention and acute interventions. Instead, adopting a geriatric medicine perspective and an individualized, person-centered approach has been suggested, including consideration of frailty (12). Frailty is a clinically identifiable state of physiological vulnerability, characterized by decreased reserves and reduced tolerance to stressor events (13). It has become more common in the aging population, and in a systematic review from 2021, the reported prevalence reached 51% among those aged 90 years or older (14). Limited attention has been paid to frailty in stroke care, but some studies have shown that the prevalence of frailty is doubled among persons with a history of stroke compared to those without. Individuals with frailty, both prior to and following a stroke, are associated with reduced quality of life, a poorer prognosis in terms of length of hospital stay, a need for nursing home care after discharge, and overall, a lower chance of recovery (15, 16). Furthermore, as pre-stroke frailty is associated with less favorable outcomes following thrombolysis and endovascular treatment (EVT), frailty should be considered when making treatment decisions both in the acute and rehabilitation phases (17, 18).

Another challenge is that treatment guidelines often rely on randomized controlled trials that frequently exclude older adults with severe stroke impairments. As a result, stroke in this age group has been studied mainly through retrospective analyses, and our knowledge and understanding of the clinical features and outcomes remain limited.

In this study, we aimed to prospectively evaluate clinical characteristics, outcomes, and the impact of frailty on stroke in people aged ≥85 years.

## 2 Methods

### 2.1 Study population

This cohort study consecutively included people aged 85 years and older with acute ischemic or hemorrhagic stroke who received standard stroke treatment (19) in the stroke unit at Karlstad Central Hospital, Sweden from April 2021 to June 2024. The unit is a primary stroke center serving a population of nearly 200,000 and has 20 beds, and acute stroke management is combined with multidisciplinary rehabilitation, such as early supported discharge. Approximately 500 patients/year are processed as “Code stroke alerts,” intravenous thrombolysis is administered either directly in the radiology department or in the stroke unit, and patients eligible for thrombectomy are transferred to Örebro University Hospital, located 110 km away. The assessments in the present study were conducted 2–7 days after admission and within 7 days of stroke onset. However, some patients with severe, fatal strokes were included later after obtaining consent from their relatives. A follow-up visit took place after 3 months.

### 2.2 Stroke-related evaluations

The stroke diagnosis was based on the World Health Organization (WHO) criteria (20) and on a combination of clinical and radiological assessment (21). All patients underwent standardized investigations at admission, such as a cerebral computed tomography (CT), a 12-lead electrocardiogram (ECG), and routine blood tests (19). Patients with ischemic stroke had cardiac telemetry for 24–48 h, and vascular evaluation was performed with carotid ultrasonography and or computed tomography angiography (CT-A). Moreover, cerebral magnetic resonance imaging (MRI) and echocardiography were carried out depending on clinical presentation. Ischemic strokes were classified etiologically using the trial of Org 10,172 Acute Stroke Treatment (TOAST) criteria (22), and in addition, the Oxfordshire Community Stroke Project (OCSP) (23) was used to classify stroke syndrome and severity. Causes of hemorrhagic stroke were determined using SMASH-U, defined as structural lesion (S), medication (M), amyloid angiopathy (A), systemic disease (S), hypertension (H), or undetermined (U) (24).

### 2.3 Assessment of pre-stroke function and outcome

The modified Rankin scale (mRS) (25) and the Barthel index (BI) (26) were used to assess disability and dependence pre-stroke, at discharge, and at 3 months post-stroke. Poor outcome was defined as an mRS of ≥4 due to a high proportion of persons classified as an mRS of 3 pre-stroke. BI was dichotomized into 75–100 and ≤70 in the analyses since BI score ≤ 70 corresponds to mRS ≥ 4 (27). A Sankey diagram to visualize functional trajectories according to mRS was created using Flourish (28). The severity of stroke was assessed using the National Institutes of Health Stroke Scale (NIHSS) (29) on admission, at discharge, and at follow-up. An NIHSS score of ≥15 was defined as severe stroke according to the NeuroARC definitions (30). Pre-stroke frailty and frailty at 3 months were estimated using the Clinical Frailty Scale (CFS) (31). CFS ≥ 5 and CFS ≥ 7 were defined as frailty and severe frailty, respectively.

### 2.4 Three-month follow-up visit

The follow-up was conducted either at the outpatient clinic, in the patient’s home, in the nursing home, or over the phone in instances where an in-person consultation was not feasible.

### 2.5 Data collected

Baseline characteristics were recorded, including age, sex, living situation, vascular risk factors (history of diagnosis and treatment), comorbidities, pre-stroke medical treatments, blood pressure at admission, body mass index (BMI), data on acute reperfusion treatment, medical complications, and dysphagia. Dysphagia screening was performed by stroke nurses using a standardized swallowing assessment tool, and in complex or unclear cases, additional investigations were carried out by speech and language therapists.

### 2.6 Statistics

Descriptive statistics were presented as counts and frequencies for categorical variables and as means and standard deviations (SD) and medians and interquartile range (IQR) for continuous variables. Predictors of death and poor functional outcome were calculated using logistic regression analyses. Univariate analyses were first conducted for selected baseline variables, some of which were dichotomized (age, living situation, NIHSS, Barthel Index, CFS, and eGFR). Age, sex, and variables with a *p*-value of <0.05 in unadjusted models were entered into a logistic multivariable regression model. Results are expressed with odds ratio (OR) with 95% confidence interval (CI). A p-value of <0.05 was considered statistically significant. Analyses were performed using the Statistical Package for Social Sciences (SPSS), version 28.0.

### 2.7 Ethical consideration

The study was approved by the Swedish Ethical Review Authority (reference number: 2020–01709). Informed consent was obtained from all patients or their next of kin when patients were unable to provide consent themselves. In some fatal cases (*n* = 14), due to ethical reasons, informed consent was provided after the hospital stay. We judged that any potential burden of study participation might be outweighed by a thorough follow-up at 3 months.

## 3 Results

**Figure 1:**
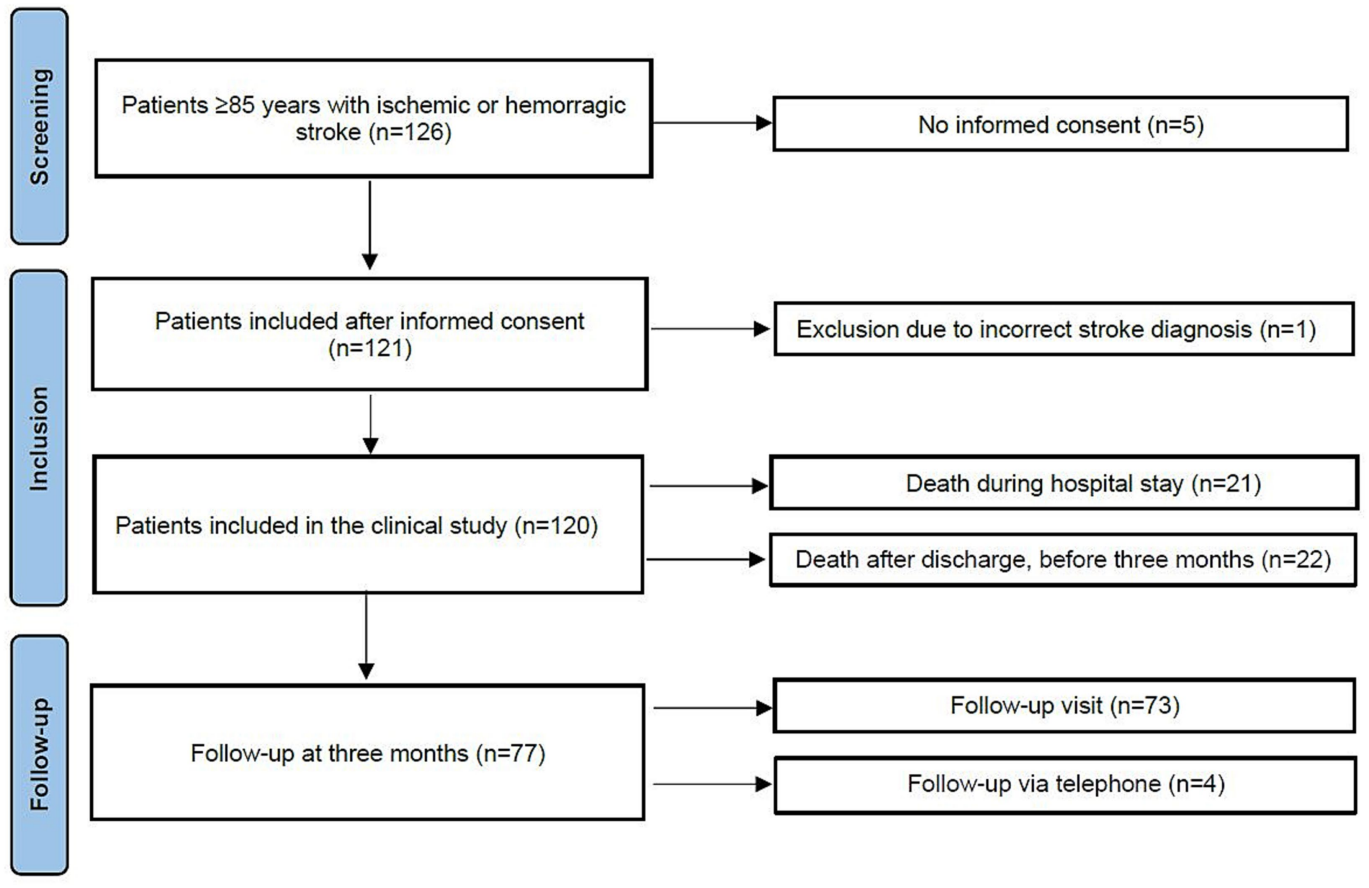
Inclusion and follow-up of the study participants.

More than 90% of patients with acute stroke eligible for this study were treated in the stroke unit (32). Of the 120 included patients, 77 survived for 3 months. A total of 73 patients had a follow-up visit, whereas four of the patients only had a follow-up via telephone with next-of-kin or other caregivers (Figure 1), with assessments including the mRS, the BI, and CFS.

The mean age was 89.1 years, and 69 (57.5%) were women. Baseline characteristics are shown in Table 1. Ischemic stroke accounted for 90.8%. Hypertension was present in 85.8% of the patients and atrial fibrillation was known in 40.8% prior to admission; after a completed stroke evaluation, atrial fibrillation was diagnosed in 55.8% of the patients. Twenty-five percent of all patients had ongoing treatments with anticoagulants and an additional 11.7% had discontinued anticoagulant treatment for various reasons despite a clear indication. Cardioembolic disease was the identified etiology in 42.2%, small vessel disease in 19.3%, large vessel disease in 11.0%, and undetermined cause in 25.7% of patients. The cases with undetermined causes included the following: no identified etiology despite evaluation in 11.0%, incomplete evaluation in 10.1% and more than one cause in 4.6% of patients. Among the 11 patients with hemorrhagic stroke, four cases were caused by hypertensive vasculopathy, one by cerebral amyloid angiopathy, four were related to anticoagulant therapy, and two had an undetermined cause. During the hospital stay, medical complications were observed in 34.2% and dysphagia in 39.2% of the patients. The median duration of the hospital stay was 7 days.

**Table 1:** Baseline characteristics of 120 persons with ischemic and hemorrhagic stroke aged ≥85 years.

Age, years, mean ±SD, median (IQR)	89.1 ± 3.5, 88 (87–91)
Female sex *n* (%)	69 (57.5)
Ischemic stroke	109 (90.8)
Hemorrhagic stroke	11 (9.2)
Co-morbidities	
Hypertension	103 (85.8)
Atrial fibrillation*	67 (55.8)
Heart failure	45 (37.5)
Ischemic heart disease	42 (35.0)
Previous stroke/TIA	40 (33.3)
Hypercholesterolemia	37 (30.8)
Diabetes	31 (25.8)
Peripheral artery disease	4 (3.3)
Current and previous smoking	49 (40.8)
Dementia	20 (16.7)
Renal impairment	14 (11.7)
Medical treatment on admission *n* (%)	
Antithrombotics	30 (25.0)
Anticoagulation (ongoing)	30 (25.0)
Anticoagulation discontinued	14 (11.7)
Antihypertensive treatment	100 (83.3)
Statins	36 (30.0)
Other admission data	
BMI, kg/m^2^, mean ±SD	24.3 ± 3.0
eGFR, ml/min, mean ±SD	50.5 ± 15.5
SBP/DBP, mmHg, mean ±SD	156.7 ± 26.5/82.8 ± 16.2
TOAST classification *n* (%)	
Cardioembolic disease	46 (42.2)
Small vessel disease	21 (19.3)
Large vessel disease	12 (11.0)
Other determined	2 (1.8)
Undetermined	28 (25.7)
OCSP *n* (%)	
Total anterior circulation infarct	14 (12.8)
Partial anterior circulation infarct	48 (44.0)
Posterior circulation infarct	22 (20.2)
Lacunar infarct	22 (20.2)
Undetermined	3 (2.8)
Medical complications *n* (%)	41 (34.2)
Urinary infection	14 (11.7)
Falling	8 (6.7)
Pneumonia	7 (5.8)
Gastrointestinal bleeding	7 (5.8)
Myocardial infarction, acute heart failure, or venous thromboembolism	8 (6.7)
Dysphagia	47 (39.2)
Duration of hospital stay, days, median (IQR)	7 (4–13)

* Including previous known atrial fibrillation and atrial fibrillation diagnosis after stroke evaluation. SD, standard deviation; IQR, interquartile range; TIA, transient ischemic attack; BMI, body mass index; eGFR, estimated glomerular filtration rate; SBP, systolic blood pressure; DBP, diastolic blood pressure; TOAST, trial of ORG 10172 in acute stroke treatment; OCSP, Oxfordshire community stroke project classification.

In total, 55.8% of the patients were identified by prehospital emergency care and processed as “Code Stroke Alerts.” Reperfusion treatments were given to 26.6% of the patients with ischemic stroke, including intravenous thrombolysis to 10.1%, EVT to 12.8%, or both therapies to 3.7% of patients. The median door-to-needle time (DNT) was 33 min and the median time from stroke onset to groin puncture was 3 h 55 min.

### 3.1 Pre-stroke function and outcomes

Of the 120 patients, 21 (17.5%) died during the hospital stay, and an additional 22 (18.3%) died within 3 months after discharge, resulting in a 3-month mortality rate of 35.8%. In-hospital death was caused by severe stroke, leading to transition to palliative care in all but one patient, who died from severe constipation. After discharge, out of 22 deaths, 16 were related to the present cerebrovascular event, 3 due to recurrent ischemic stroke, and 3 due to other causes. Dysphagia was present in most of the patients who died during the hospital stay, 20 out of 21 patients, and in 14 out of 22 patients who died after discharge.

**Figure 2:**
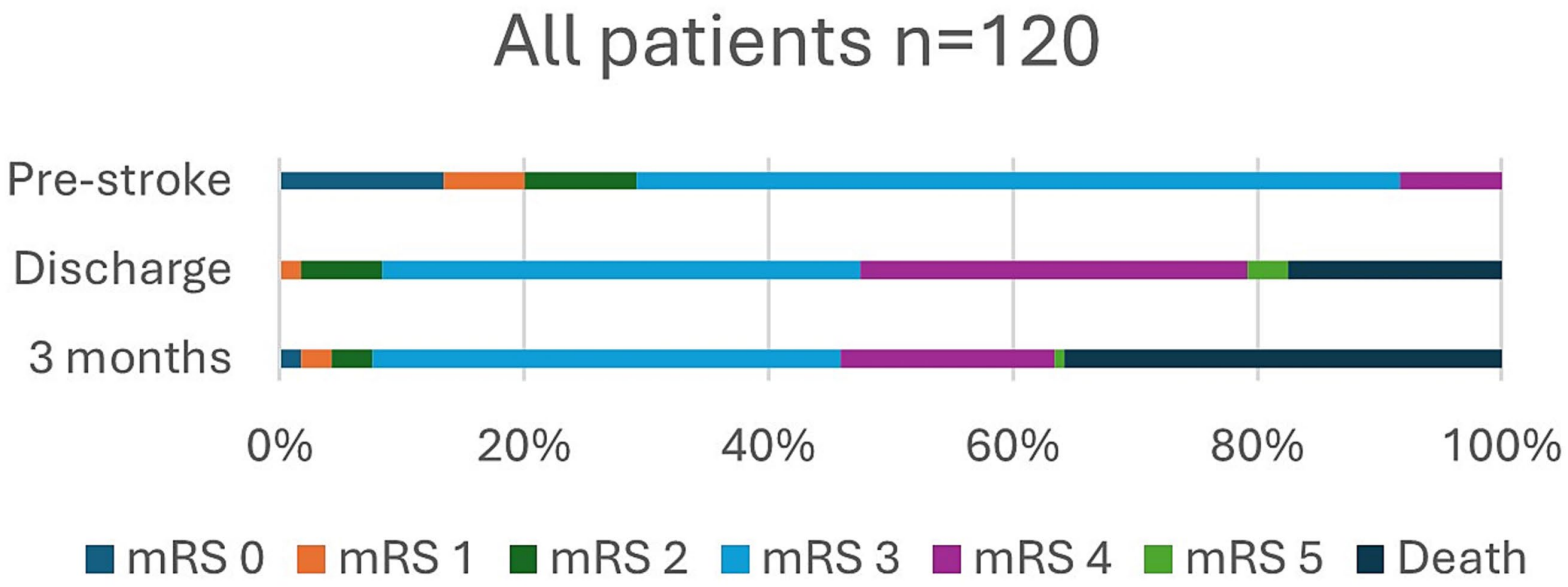
Modified Rankin Scale (mRS), pre-stroke, at discharge, and at 3 months.

**Figure 3:**
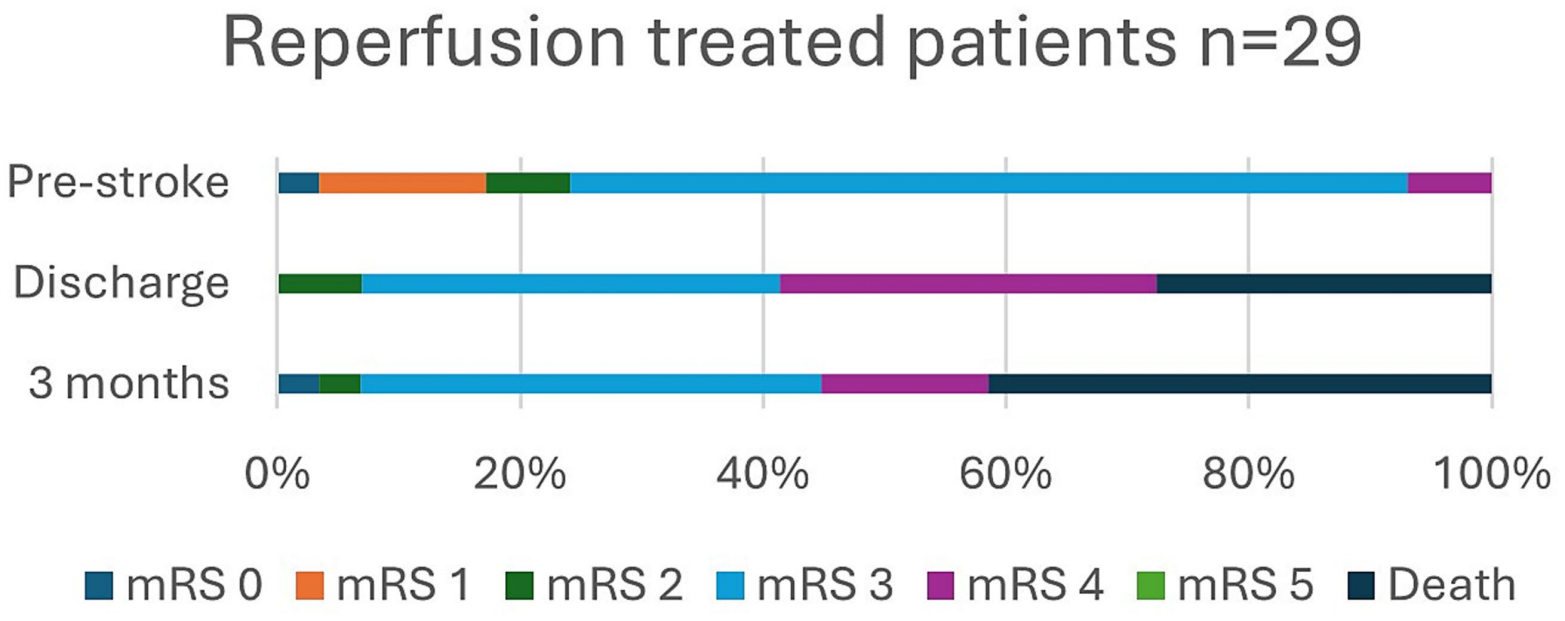
Modified Rankin Scale (mRS), pre-stroke, at discharge, and at 3 months in reperfusion treated patients.

**Figure 4:**
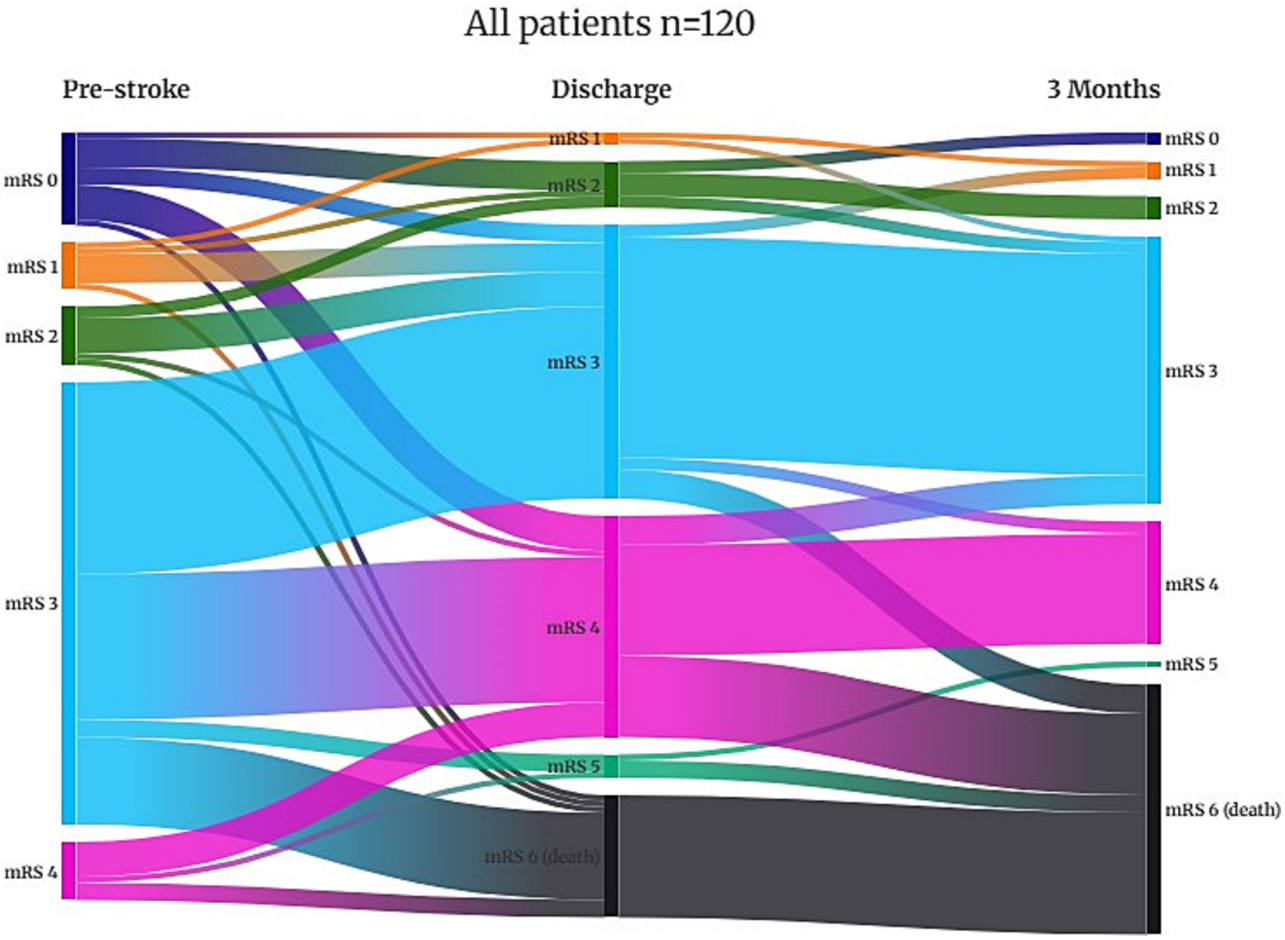
Functional trajectories according to the modified Rankin Scale (mRS).

**Figure 5:**
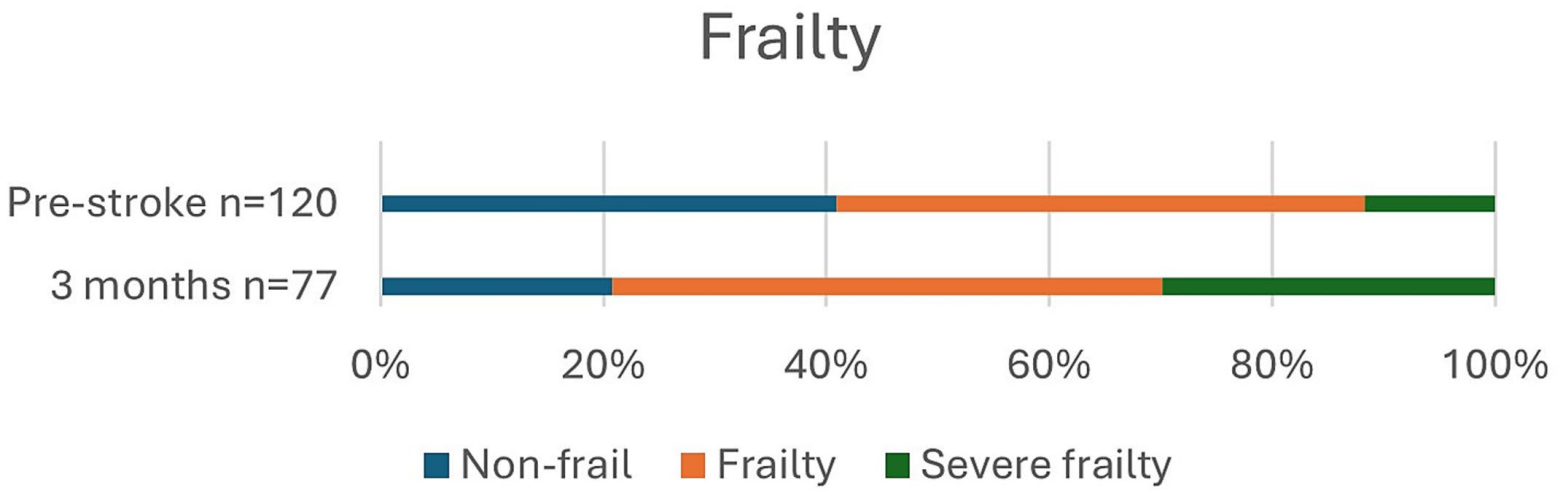
Frailty pre-stroke and at 3 months.

In Table 2, data are shown to include living situation, degree of disability (BI and mRS), frailty (CFS) prior to and after stroke, and NIHSS score on admission, at discharge, and 3 months for all 120 patients and separately for those treated with reperfusion. In addition, mRS, including variability in mRS scores over time, and frailty are presented in Figures 2–5.

**Table 2:** Pre-stroke function and clinical outcomes in 120 patients, aged ≥ 85 years, with ischemic and hemorrhagic stroke including separate data on 29 reperfusion treated patients, *N* (%).

Variables	Pre stroke	At discharge	At 3 months
All patients	*n* = 120	*n* = 99	*n* = 77
Living situation			
Nursing home or homecare >3/day	27 (22.6)	53 (53.5)	36 (46.8)
Death		21 (17.5)	43 (35.8)
mRS 0–2	35 (29.2)	10 (8.3)	9 (7.5)
mRS 3	75 (61.7)	47 (39.2)	46 (38.3)
mRS ≥ 4	10 (9.1)	63 (52.5)	65 (54.2)
Barthel index <75	18 (15.0)	58 (59.8)	27 (35.1)
Frailty CFS ≥ 5	72 (59.5)		61 (79.2)
Severe frailty CFS ≥ 7	14 (11.7)		23 (29.9)
NIHSS median (IQR)	6 (2–15) on admission	3 (1–6)	2 (1–4)
Reperfusion-treated patients	*n* = 29	*n* = 21	*n* = 17
Living situation			
Nursing home or homecare >3/day	8 (27.5)	11 (52.4)	10 (58.8)
Death		8 (27.6)	12 (41.4)
mRS 0–2	7 (24.1)	2 (6.9)	2 (6.9)
mRS 3	20 (69.0)	10 (34.5)	11 (37.9)
mRS ≥ 4	2 (6.9)	17 (58.6)	16 (55.2)
Barthel index <75	4 (13.8)	15 (71.4)	7 (41.2)
Frailty CFS ≥ 5	18 (62.1)	20 (95.2)	15 (88.2)
Severe frailty CFS ≥ 7	3 (10.3)	14 (66.7)	5 (29.4)
NIHSS median (IQR)	13 (5.5–19.5) on admission	4 (2–10)	4 (2–10)

mRS, modified rankin scale; CFS, Clinical Frailty Scale; IQR, interquartile range.

Prior to stroke, 22.6% of the patients lived in a nursing home or received home care more than three times daily, and it had increased to 46.8% at 3 months. Independence (mRS 0–2) was observed in 29.2% of patients pre-stroke and in only 7.5% of patients at 3 months post-stroke. Pre-stroke, 90.9% of the patients had an mRS score of 0–3 since most patients received some degree of home care. At 3 months, an mRS score of 0–3, which indicates a good outcome, was achieved in 45.8% of the patients. Overall, in 32.5% of patients, mRS was unchanged at 3 months compared to prior to stroke. Frailty, with CFS ≥ 5, was observed in 59.5% of patients pre-stroke and in 79.2% at 3 months, and severe frailty, with CFS ≥ 7, was observed in 11.7% of patients before stroke and in 29.9% at follow-up.

Median NIHSS on admission was 6 (2–15) for all patients and 13 (5.5–19.5) for patients receiving reperfusion therapy.

### 3.2 Predictors of unfavorable outcome and mortality

In the adjusted regression models (Tables 3, 4), pre-stroke BI of ≤70, NIHSS score of ≥15 on admission, and post-stroke dysphagia were significantly associated with death at 3 months. In patients with ischemic stroke, 3-month mortality was correlated with higher age (≥89 years), total anterior circulation infarct (TACI), pre-stroke BI of ≤70, and dysphagia. Poor outcome, i.e., mRS ≥ 4, was, for all patients, significantly associated with higher age, pre-stroke severe frailty (CFS ≥ 7), and dysphagia. In addition, a poor outcome was observed in all ischemic stroke patients with TACI. Overall, the strongest association was observed for dysphagia, with OR of 15.97 (95% CI, 5.46–46.67), for the entire study sample.

**Table 3:** Predictors of death at 3 months among older persons, ≥85 years, with stroke.

Ischemic stroke, *n* = 109					Ischemic and hemorrhagic stroke, *n* = 120			
Characteristics	Unadjusted OR (95% CI)	*p*-value	Adjusted OR (95% CI)	*p*-value	Unadjusted OR (95% CI)	*p*-value	Adjusted OR (95% CI)	*p*-value
Male sex	0.78 (0.35–1.77)	0.560			0.83 (0.39–1.77)	0.624		
Age ≥89 years*	2.97 (1.28–6.89)	**0.011**	3.99 (1.19–13.41)	**0.025**	2.40 (1.12–5.14)	**0.025**		
Atrial fibrillation	1.82 (0.79–4.18)	0.161			1.82 (0.84–3.93)	0.128		
Heart failure	0.71 (0.30–1.67)	0.433			0.84 (0.39–1.83)	0.658		
Previous Stroke/TIA	1.31 (0.56–3.05)	0.530			1.54 (0.70–3.36)	0.283		
ADL pre-stroke								
Barthel ≤70*	7.00 (2.01–24.34)	**0.002**	10.01 (1.83–54.89)	**0.008**	6.24 (2.04–19.05)	**0.001**	5.30 (1.25–22.51)	**0.024**
Nursing home/ homecare >3/d*	2.70 (1.06–6.85)	**0.037**			2.90 (1.21–6.99)	**0.018**		
Frailty								
CFS ≥ 7*	3.45 (1.01–11.79)	**0.048**			3.81 (1.19–12.24)	**0.025**		
mRS 4–5*	2.26 (0.53–9.62)	0.271			2.96 (0.79–11.14)	0.109		
NIHSS on admission ≥15*	14.95 (3.87–57.70)	**<0.001**			16.76 (4.52–62.24)	**<0.001**	8.14 (1.90–34.87)	**0.005**
eGFR <45 on admission*	1.66 (0.72–3.82)				1.54 (0.70–3.36)	0.283		
Dysphagia	11.36 (4.41–29.23)	**<0.001**	4.94 (1.48–16.53)	**0.009**	12.02 (4.95–29.20)	**<0.001**	6.23 (2.34–16.62)	**<0.001**
TOAST								
Cardioembolic disease*	0.68 (0,30–1.54)	0.355			**-**	**-**		
OCSP								
TACI*	46.80 (5.77–379.67)	**<0.001**	14.59 (1.48–143.78)	**0.022**	**-**	**-**		
Acute intervention								
Reperfusion	1.75 (0.72–4.23)	0.215			**-**	**-**		
Only intravenous thrombolysis	0.53 (0.19–1.48)	0.225			**-**	**-**		
Thrombectomy	1.90 (0.68–5.33)	0.225			**-**	**-**		

Univariate and adjusted odds ratios.

TIA, transient ischemic attack; ADL, activities of daily living; CFS, clinical frailty scale; mRS, modified Rankin scale; NIHSS, national institute of health stroke scale; eGFR, estimated glomerular filtration rate; TOAST, trial of ORG 10172 in acute stroke treatment, OCSP, Oxfordshire community stroke project classification; TACI, total anterior circulation infarction. * Dichotomized variables: Age (85-88y/≥89y), Barthel (75–100/≤70), Living situation (Nursing home or homecare >3/d/ No home care or ≤3/d), Frailty, CFS (0–6/≥7), mRS (0–3/4–5), NIHSS (0–14/≥15), eGFR (≥45/0–44), TOAST (cardioembolic disease/ all other etiologies), OCSP (TACI/ all other classifications). Bold values: *p* < 0.05.

**Table 4:** Predictors of poor outcome, mRS ≥ 4 at 3 months among older persons, ≥85 years, with stroke.

Ischemic stroke, *n* = 109					Ischemic and hemorrhagic stroke, *n* = 120			
Characteristics	Unadjusted OR (95% CI)	*p*-value	Adjusted OR (95% CI)	*p*-value	Unadjusted OR (95% CI)	*p*-value	Adjusted OR (95% CI)	*p*-value
Male sex	0.62 (0.29–1.32)	0.215			0.61 (0.29–1.26)	0.180		
Age ≥89 years*	3.81 (1.72–8.43)	**<0.001**	6.63 (2.18–20.19)	**<0.001**	2.89 (1.37–6.12)	**0.005**	3.82 (1.45–10.06)	**0.007**
Risk factors								
Atrial fibrillation	1.03 (0.49–2.20)	0.932			1.10 (0.53–2.27)	0.794		
Heart failure	0.60 (0.28–1.32)	0.207			0.71 (0.34–1.50)	0.369		
Previous Stroke/TIA	1.61 (0.72–3.60)	0.250			1.67 (0.77–3.62)	0.197		
Living situation pre stroke								
Nursing home/ homecare >3/d*	3.00 (1.13–7.99)	**0.028**			3.05 (1.18–7.90)	**0.022**		
Frailty								
CFS ≥ 7*	5.78 (1.20–27.77)	**0.029**	11.42 (1.59–81.88)	**0.015**	6.00 (1.28–28.12)	**0.023**	7.27 (1.14–46.26)	**0.036**
NIHSS on admission ≥15*	5.82 (1.55–21.85)	**0.009**			6.85 (1.88–24.95)	**0.004**		
eGFR <45 on admission*	1.58 (0.72–3.51)	0.257			1.43 (0.66–3.08)	0.365		
Complications								
Dysphagia	14.00 (5.09–38.45)	**<0.001**	18.82 (5.72–61.90)	**<0.001**	13.95 (5.20–37.40)	**<0.001**	15.97 (5.46–46.67)	**<0.001**
TOAST								
Cardioembolic disease*	1.03 (0.48–1.21)	0.935						
Acute intervention								
Reperfusion therapy, overall	1.29 (0.55–3.04)	0.554			-	-		
Only intravenous thrombolysis	0.78 (0.28–2.16)	0.636			-	-		
Thrombectomy	1.28 (0.46–3.53)	0.636			-	-		

Univariate and adjusted odds ratios.

TIA, transient ischemic attack; CFS, clinical frailty scale; NIHSS, National Institute of Health Stroke Scale; eGFR, estimated glomerular filtration rate; TOAST, trial of ORG 10172 in acute stroke treatment. * Dichotomized variables: Age (85-88y/≥89y), Living situation (Nursing home or homecare >3/d/ No home care or ≤3/d), Frailty, CFS (0–6/≥7), NIHSS (0–14/≥15), eGFR (≥45/0–44), TOAST (cardioembolic disease/ all other etiologies). Bold values: *p* < 0.05.

## 4 Discussion

In the present study, a good functional outcome was achieved in almost half of the patients, despite a mortality of 36% at 3 months. We found that higher age, low pre-stroke function, severe frailty prior to stroke, TACI, high NIHSS scores on admission, and post-stroke dysphagia were predictors of death or poor outcome.

A median NIHSS score of 6 on admission, as reported in this study, is substantially higher than the overall median NIHSS score of 3 in the entire Swedish stroke population (32). The higher NIHSS score is most likely related to the high prevalence of cardioembolic stroke and pre-stroke comorbidity in this cohort. Previous research has shown that cardioembolic disease, due to atrial fibrillation, is associated with intracranial large vessel occlusion, more severe stroke, poor recovery, and higher mortality compared to other etiologies (33). Atrial fibrillation increases with age, reaching a prevalence of nearly one in four women aged 90 years or older (34), and was found in two-thirds of endovascular-treated nonagenarians in a study from the German Stroke Registry (35). In our study, the prevalence of atrial fibrillation was 56%, although probably even higher, since, due to ethical reasons, in patients with severe stroke, confusional state, and transition to palliative care, etiological investigations were restricted. In 10 of the 28 patients with undetermined etiology, atrial fibrillation was present, but the etiology was still unclear due to multiple potential causes or to limited vascular evaluation, and in three patients, no cardiac monitoring was performed.

Given the highly preventive effect of anticoagulation, identifying patients with atrial fibrillation is of utmost importance. Despite an increasing use of anticoagulation (36), it is probably still underutilized in many countries (37). Moreover, discontinuation of treatment is more common in older people (38). In the present study, 44 of 49 patients with known atrial fibrillation were prescribed anticoagulation, of whom 14 had no ongoing active treatment due to poor adherence in 4 patients and adverse events or surgical procedures in the remaining 10. A recent study demonstrated that discontinuation of anticoagulation doubled the risk of recurrent ischemic stroke in patients with atrial fibrillation compared to those who maintained their treatment (39). Considering the complexity in older patients with atrial fibrillation, including bleeding risks, comorbidities, and frailty, adopting a comprehensive geriatric- and person-centered approach with in-depth medication review and evaluation of the individual’s functional level is likely to be beneficial to reduce discontinuation when possible and optimize overall anticoagulation treatment (40).

In addition to well-known predictors, such as severe stroke and higher age, we found that reduced ADL function prior to stroke was a determinant of death at 3 months, consistent with results from previous studies (4, 6). On the other hand, older people are heterogenous and several individuals remain autonomous and healthy. In the present study, 29% were fully independent with an mRS score of 0–2 pre-stroke as compared to 85% in the whole Swedish stroke population (32). However, another 29% were almost independent with an mRS of 3, receiving home care less than once a day. Still, the latter group, requiring minimal support, is classified as mRS 3, reflecting the scale’s limitations, since the degree of disability and need for assistance may differ substantially among patients within the mRS 3 group.

Despite a mean age of 89 years and a high prevalence of pre-stroke dependence and comorbidities, almost 27% of the patients with ischemic stroke received reperfusion treatment in the present study compared to 19% among all patients with ischemic stroke in Sweden (32). This finding is likely attributable to frequent severe stroke presentations and a well-functioning stroke alarm pathway, resulting in over half of the patients being categorized as “Code Stroke Alerts” after their stroke onset. Among reperfusion-treated patients, 20 of 29 were classified as an mRS score of 3 pre-stroke. Good functional outcome, with an mRS score of 0–3, at 3 months was achieved in 13 of 29 patients, including 8 of 18 patients receiving EVT. Older individuals or those with reduced ADL-function pre-stroke have largely been excluded from randomized controlled studies in stroke research. In a meta-analysis of seven RCTs on EVT, 77 out of 1,764 patients were ≥85 years old and only 46 patients were dependent pre-stroke with an mRS of ≥2 (41). An overall worse functional outcome at 90 days was found in older persons compared to the younger ones; however, a benefit of EVT was observed in patients ≥ 85 years compared to conservative management (41). Furthermore, retrospective studies have shown that older people with pre-stroke functional impairments, a good outcome defined as unchanged pre-stroke state, may benefit from EVT (35, 42, 43). In addition to a high prevalence of pre-stroke functional impairment, more procedural difficulties, comorbidities, and post-stroke complications negatively affect prognosis in older people (44).

Due to the heterogeneity in older people’s biological age, a careful individualized selection is needed to decide whether to offer reperfusion treatment as well as other advanced medical or rehabilitation interventions in people with stroke. These decisions should be based on an assessment of biological and not chronological age (35, 42–45). As a marker of biological aging, frailty is increasingly used in clinical practice but has not had a prominent role in stroke care (18). Indicators of frailty are sarcopenia, weight loss, reduced gait speed, and fatigue. Sarcopenia, a progressive loss of muscle mass and strength, due to aging, malnutrition, and physical inactivity is associated with worse stroke outcomes (46). Assessment of frailty pre- and post-stroke has been suggested to support the decision-making and may potentially add important prognostic information beyond routine assessments with mRS and NIHSS (16–18). The need for evaluation and management of frailty is a growing concern as the number of old and frail individuals is increasing. The prevalence of pre-stroke frailty in our study was nearly 60% and severe frailty prior to stroke was an independent predictor of poor outcome at 3 months. These results are in line with those of previous studies showing an association between frailty and poor outcome in stroke (16–18). Patients with severe or very severe frailty, where the individual is in an irreversible state and often approaching death, should in most cases not be offered endovascular or other advanced interventions routinely, whereas people with mild or moderate frailty seem to have a potential for reversing functional impairments and, therefore, should receive the same treatment as robust patients without multimorbidity or reduced functional level, independently of chronological age (47, 48). In the current study, medical complications were observed in 34.2% of patients. The most common were urinary tract infection (11.7%), falls (6.7%), and myocardial infarction, acute heart failure, or venous thrombosis (6.7%). Pneumonia was only diagnosed in 5.8% of the patients, which may be explained not only by systematic assessment of dysphagia and very early mobilization but also by the high in-hospital mortality directly related to the stroke event.

Dysphagia, associated with frailty (49), is a common and challenging problem after stroke and was observed in 39% of the patients in the present study. Consistent with the literature, we found a strong correlation between dysphagia and poor outcome and mortality at 3 months (50). Early swallowing difficulties following stroke may improve, but improvement is less likely in older adults with larger strokes, pre-stroke frailty, and cognitive impairments (18, 51). If persistent dysphagia is present, an individualized, careful multidisciplinary evaluation is required before a decision regarding long-term enteral feeding is made, including the assessment of frailty and overall prognosis (18). Pre-stroke frailty may increase mortality in patients receiving percutaneous endoscopic gastrostomy (PEG) tube (52), and in most cases with severe frailty, PEG should probably be avoided.

### 4.1 Strengths and limitations

The strengths of this study are the consecutive and high proportion of inclusion among screened patients and the study of a “real world” old stroke population. However, there are some limitations of this study. First, approximately 10% of the patients with stroke were treated in other departments and therefore not eligible for the study. This may partly be due to atypical clinical presentations in some older adults, such as acute delirium and falls, which can result in an initial misdiagnosis and subsequent management outside the stroke unit. Second, etiological investigations were sometimes limited due to cognitive impairments, affected consciousness, and lack of patient co-operation. Finally, this is a one-center study, with a relatively small sample size that could limit the generalizability.

## 5 Conclusion

We confirmed a high prevalence of cardioembolic disease in persons aged 85 years or older with stroke, highlighting the importance of the detection of atrial fibrillation and optimizing anticoagulant treatment. Moreover, severe frailty prior to stroke and post-stroke dysphagia were independent predictors of poor outcome. Assessment of frailty may be beneficial in decision-making regarding both acute interventions, rehabilitation, and the direction of care. Considering the demographic shift, future stroke research should focus on older people, including those with pre-stroke frailty and functional impairments.

## Data Availability

The original contributions presented in the study are included in the article/supplementary material, further inquiries can be directed to the corresponding authors.
